# Bioinformatics analysis identified CDC20 as a potential drug target for cholangiocarcinoma

**DOI:** 10.7717/peerj.11067

**Published:** 2021-03-17

**Authors:** Prin Sungwan, Worachart Lert-itthiporn, Atit Silsirivanit, Nathakan Klinhom-on, Seiji Okada, Sopit Wongkham, Wunchana Seubwai

**Affiliations:** 1 Biomedical Science Program, Graduate School, Khon Kaen University, Khon Kaen, Thailand; 2 Cholangiocarcinoma Research Institute, Khon Kaen University, Khon Kaen, Thailand; 3 Department of Biochemistry, Faculty of Medicine, Khon Kaen University, Khon Kaen, Thailand; 4 Division of Hematopoeisis, Joint Research Center for Human Retrovirus Infection, Kumamoto University, Kumamoto, Japan; 5 Department of Forensic Medicine, Faculty of Medicine, Khon Kaen University, Khon Kaen, Thailand

**Keywords:** Bioinformatic analysis, Cholangiocarcinoma, GEO, TCGA, Spheroid, In silico, CDC20, Dinaciclib

## Abstract

**Background:**

Cholangiocarcinoma (CCA) is a malignancy that originates from bile duct cells. The incidence and mortality of CCA are very high especially in Southeast Asian countries. Moreover, most CCA patients have a very poor outcome. Presently, there are still no effective treatment regimens for CCA. The resistance to several standard chemotherapy drugs occurs frequently; thus, searching for a novel effective treatment for CCA is urgently needed.

**Methods:**

In this study, comprehensive bioinformatics analyses for identification of novel target genes for CCA therapy based on three microarray gene expression profiles (GSE26566, GSE32225 and GSE76297) from the Gene Expression Omnibus (GEO) database were performed. Based on differentially expressed genes (DEGs), gene ontology and pathway enrichment analyses were performed. Protein-protein interactions (PPI) and hub gene identifications were analyzed using STRING and Cytoscape software. Then, the expression of candidate genes from bioinformatics analysis was measured in CCA cell lines using real time PCR. Finally, the anti-tumor activity of specific inhibitor against candidate genes were investigated in CCA cell lines cultured under 2-dimensional and 3-dimensional cell culture models.

**Results:**

The three microarray datasets exhibited an intersection consisting of 226 DEGs (124 up-regulated and 102 down-regulated genes) in CCA. DEGs were significantly enriched in cell cycle, hemostasis and metabolism pathways according to Reactome pathway analysis. In addition, 20 potential hub genes in CCA were identified using the protein-protein interaction (PPI) network and sub-PPI network analysis. Subsequently, CDC20 was identified as a potential novel targeted drug for CCA based on a drug prioritizing program. In addition, the anti-tumor activity of a potential CDC20 inhibitor, namely dinaciclib, was investigated in CCA cell lines. Dinaciclib demonstrated huge anti-tumor activity better than gemcitabine, the standard chemotherapeutic drug for CCA.

**Conclusion:**

Using integrated bioinformatics analysis, CDC20 was identified as a novel candidate therapeutic target for CCA.

## Introduction

Nowadays, bioinformatics approaches have been widely used for analysis and management of data from several sources. The Gene Expression Omnibus (GEO) database and The Cancer Genomics Atlas (TCGA) offer tools for screening and discovery of novel molecular pathways, promising biomarkers, genetic alterations, prognosis and novel effective target molecules for several cancers. By this approach, several potential key genes, biomarkers and pathways involved with gastric cancer (*Yan et al., 2018)*; lung adenocarcinoma (*Guo, Ma & Zhou, 2019*) and myelodysplastic syndrome (*Le, 2019*) have been identified.

Cholangiocarcinoma is a fatal cancer arising from malignant transformation of cholangiocytes. CCA represents 3% of gastrointestinal cancers worldwide. The incidence and mortality of CCA, however, are very high in the Eastern world, especially in Southeast Asian countries (*Banales et al., 2016*). The prognosis of CCA patients is very poor, with a very low 5-year survival (Ramírez-Merino, 2013). The gold standard treatment for CCA is the surgery with R0 resection. Unfortunately, approximately 3% of CCA patients could be offered with R0 resections (Luvira et al., 2016; Adeva et al., 2019). Most CCA patients usually come with an advanced stage, in which the tumor has already spread to the secondary sites or other organs (*Blechacz, 2017*). Currently, many chemotherapy drugs have been used and reported for CCA treatment such as gemcitabine, 5-Fluorouracil (5-FU), cisplatin, sorafenib, capecitabine plus cisplatin and oxaliplatin/cetuximab (*Keating & Santoro, 2009; Adeva et al., 2019; Chen et al., 2015; Ben-Josef et al., 2015*)*.* The widely used one is gemcitabine with an approximate survival time of 21.5 months (*Murakami et al., 2009*). 5-FU also provided a poor overall response rate with 0–40% and a median survival about 2–12 months (*Thongprasert, 2005*). Resistance to this wide range of chemotherapeutic drugs and high damage to adjacent tissues often occurs in CCA patients. Thus, searching for novel target molecules or alternative approaches for an effective treatment for CCA is urgently needed. Using the bioinformatics approaches may accelerate the discovery of the potential molecules that might be a new target for CCA treatment.

In this study, we performed an in-silico analysis for screening and identification of potent novel targets for CCA treatments using three microarray datasets retrieved from the GEO database. The differential gene expression analysis was performed for the identification of differentially expressed genes (DEGs) between CCA and normal tissues. Then, Gene Ontology (GO) and Reactome pathways were analyzed. The protein-protein interaction network (PPI) and sub-PPI network analyses of common DEGs were constructed to identify the key genes associated with CCA. Subsequently, prediction and prioritizing of candidate novel anti-cancer drugs was performed. Finally, the cytotoxicity of the candidate drug target on viability of CCA cell lines in 2- and 3-dimensional (2D, 3D) cell culture models were validated to prove the concept.

## Materials & Methods

### Cell culture

Human CCA cell lines derived from CCA patient tissues that included KKU-213A (poorly differentiated squamous cell carcinoma), KKU-213B (well differentiated squamous carcinoma) (*Sripa et al., 2020*), KKU-100 (poorly differentiated adenocarcinoma) (Sripa et al., 2005), and KKU-452 (poorly differentiated adenocarcinoma) (*Saensa-ard et al., 2017*) were cultured in Dulbecco’s Modified Eagle’s Medium (DMEM)(Gibco life technologies Corporation, Grand Island, NY) with 25 mM glucose supplemented with 1% antimycotic-antibiotic and 10% heat-inactivated fetal bovine serum at 37 °C, 5% CO_2_.

For the 3D cell culture, the adherent CCA cells were trypsinized and seeded into a 96 well ultra-low attachment plates (Corning, Incorporated, Kennebunk, Maine) at the optimal starting cell numbers. CCA cells were then cultured at 37 °C, 5% CO_2_ for 24–72 h for spheroid formation. Spheroid morphology was observed under an inverted light microscope. Spheroid integrity were analyzed by ImageJ NIH software (https://imagej.nih.gov/ij/download.html).

### Microarray data sources

Three gene expression datasets of CCA and normal tissues were retrieved from the GEO database (https://www.ncbi.nlm.nih.gov/geo/) including GSE26566, GSE32225 and GSE76297. GSE26566 based on GPL6104 Illumina Humanref-8 v2.0 expression bead chip platforms, GSE32225 based on GPL8432 Illumina Humanref-8 WG-DASL v3.0 platforms and GSE76297 based on GPL17586 [HTA-2_0] Affymetrix Human Transcriptome Array 2.0 [transcript (gene) version].

### Differentially expressed gene (DEGs) analysis

The DEGs between cancer and normal tissues were analyzed using the GEO2R online tool (https://www.ncbi.nlm.nih.gov/geo/geo2r/). Genes that met the cutoff criteria, adjusted *P*-value <0.05 and —logFC—≥ 0.5, were considered DEGs. A volcano plot was performed for data visualization using RStudio (https://rstudio.com/). Moreover, Venn diagrams were plotted (http://bioinformatics.psb.ugent.be/webtools/Venn/) for elucidating common DEGs among three microarray datasets.

### Gene ontology, pathway enrichment, protein-protein interaction (PPI) and hub gene identification analysis

The common DEGs were inputted into STRING software version 11.0 (https://string-db.org/) for analyzing the interactions among various proteins to constitute a PPI network, gene ontology and pathway enrichment. Gene functions were classified into cellular components (CC), cellular processes (CP), molecular function (MF) and the Reactome pathway. GO results were visualized as a bubble plot by RStudio software. Screening and identification of hub genes were conducted with cytohubba (https://apps.cytoscape.org/apps/cytohubba) (*Chin et al., 2014*) of the Cytoscape (https://cytoscape.org/) plug-in MCC model. 

### Prediction and prioritizing of novel candidate anti-cancer drugs

After hub gene identifications, the next step was further prioritizing and predicting a novel anti-cancer drug based on up-regulated hub genes using PanDrugs software (https://www.pandrugs.org) (Piñeiro Yáñez et al., 2018). Prioritization criteria for the selection of a novel anti-tumor drug was that the candidate drug had to have been used in clinical trials or is already approved for clinical use.

### RNA extraction and real-time PCR

Cells were collected and total RNA samples were extracted from CCA cell lines using TRIzol^®^ reagent (Invitrogen; Thermo Fisher Scientific, Van Allen Way, California.) according to the manufacturer’s protocol. RNA (2 µg) was reverse transcribed to cDNA. The system conditions for real-time PCR contained (10 µl): 5 µl for 2X SYBR Green I Master LightCycler^®^480 SYBR green I master (Roche Diagnostics GmbH, Mannheim, Germany), 2 µl of cDNA (25 ng/µl), 2 µl of forward and reverse primers and 1 µl DD H_2_O PCR grade. The primers for CDC20 sequences were as follows: forward, 5′-CGGGTAGCAGAACACCATGT-3′reverse, 5′-ACTGGCCAAATGTCGTCCAT-3′. The PCR conditions were 95° for 5 min, followed by 50 cycles at 95° for 10 s and 62° for 10 s.

### Western blot analysis

Cells were lysed with RIPA buffer (Thermo Fisher Scientific, Rockford, Illinois) containing Proteinase Inhibitor Cocktail (NACALAI TESQUE, INC., Kyoto, JAPAN). The lysates were shaken at 4 °C overnight. Protein content was determined using BCA assay kit (Thermo Fisher Scientific, Rockford, Illinois). Protein lysates were separated in a 12% (for actin) or 15% (for CDC20) sodium dodecyl sulfate polyacrylamide gel electrophoresis (SDS-PAGE) and transferred on a polyvinylidene fluoride or polyvinylidene difluoride (PVDF) membrane (Merck KGaA, Darmstadt, Germany) using Towbin transfer buffer in a wet tank transfer blotting system (Bio-Rad Laboratories (Shanghai) Co., Ltd., Shanghai, China). After blocking with 5% skimmed milk for 1 h at room temperature (RT), membranes were incubated with each primary antibody, CDC20 (Cell Signaling Technology, Tokyo, Japan), or actin (Santa Cruz Biotechnology, Inc., Dallas, Texas) at 4 °C, overnight. Blots were then incubated with anti-rabbit IgG, HRP-linked Antibody (Cell Signaling Technology, Tokyo, Japan) or anti-mouse IgG, HRP-linked Antibody (Cell Signaling), at RT for 1 h. The western blot bands were detected using an ImageQuant LAS 4000 (GE Healthcare Bio-Sciences AB, Uppsala, Sweden).

### The cytotoxicity test in two-dimensional (2D) and three-dimensional (3D) cell culture models

For the 2D cell culture model, CCA cell lines were seeded into 96-well plates (1,500 cells/well) overnight (16 h) and then treated with various concentrations of gemcitabine (Sigma-Aldrich, St. Louis, Missouri) or dinaciclib (Abcam plc., Cambridge, UK) for 72 h. After incubation, cell viability was measured by ATPlite 1step (PerkinElmer, inc, Waltham, Massachusetts) for luminescence detection of ATP from cultured cells under the 2D model using SpectraMax L Microplate Reader (Molecular Devices, St, San Jose, California).

For the 3D cell culture model, 2,500 cells/well of CCA cell lines were seeded into 96 well ultra-low attachment flat-bottom plates (Corning, Incorporated, Kennebunk, Maine), and incubated for 24 to 78 h depending on cell type. The formed spheroids were then exposed to gemcitabine or dinaciclib for 72 h. Cell viability was measured by the ATPlite3D kit (PerkinElmer, inc, Waltham, Massachusetts) using SpectraMax L Microplate Reader. Cells cultured in DMSO; 0.01% for 2D-culture and 0.4% for 3-D culture, were used as a vehicle control in all drug testing experiments. Three separated experiments with triplicate assays were performed.

### Statistical analysis

Statistical analyses were conducted using Graphpad Prism software version 7.03 (GraphPad Software, Inc., San Diego, California). All experiments were performed in three independent experiments. The cytotoxicity results are expressed as mean ± SD. Statistical significance was determined by Students’ *t* test and *P* < 0.05 was noted as statistical significance.

## Results

### Identification of common DEGs

**Figure 1:**
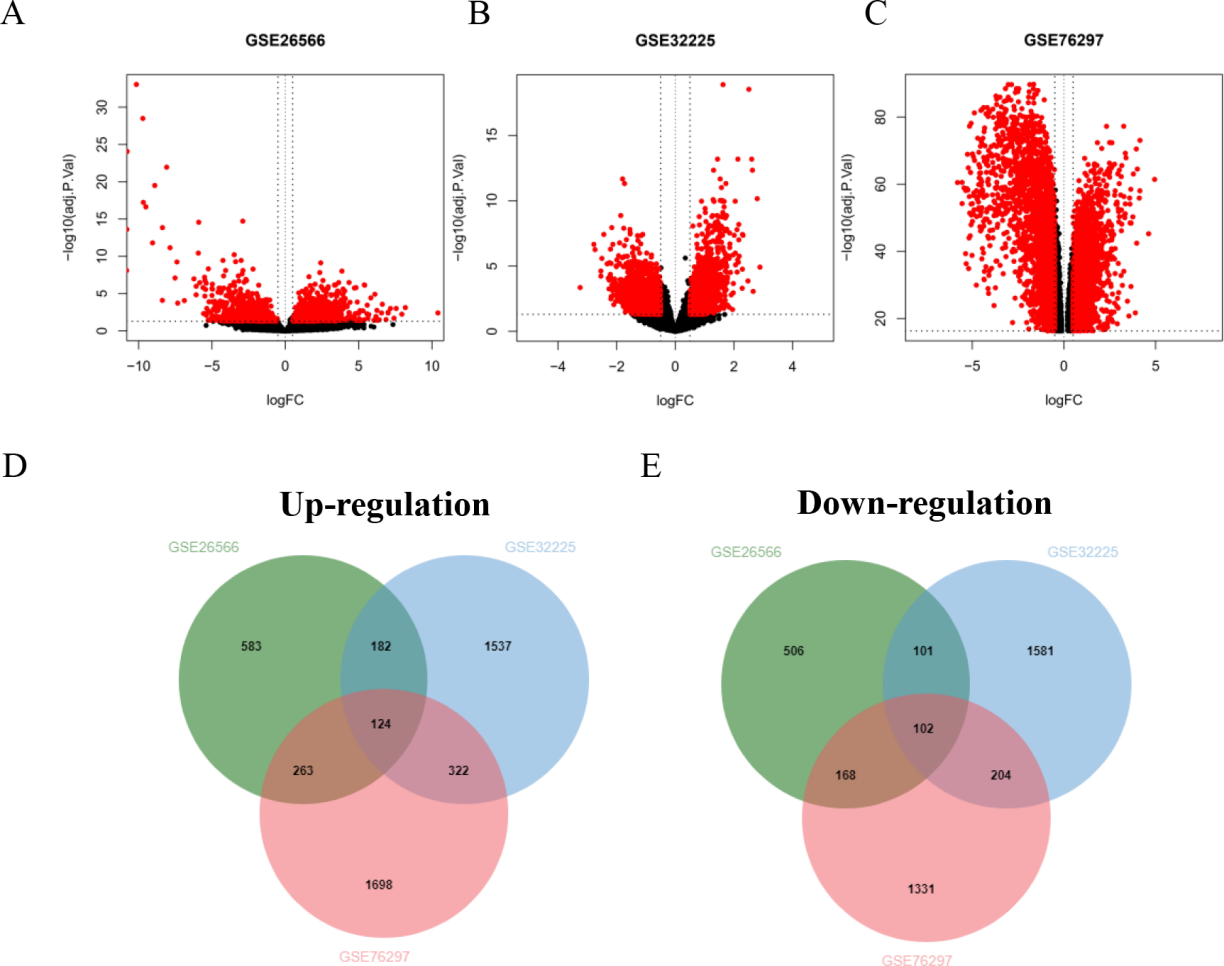
GEO dataset of differentially expressed genes (DEGs) from cholangiocarcinoma (CCA) patients. (A–C) Volcano plot of DEGs in each GEO dataset. Red represents the genes that were significantly up- or down-regulated in CCA samples. Black dots represent the genes that were not significantly up- or down- regulated in CCA samples. The dotted vertical lines indicate the significant threshold filters. Venn diagrams illustrate the number of common (D) up- and (E) down- regulated DEGs shared by the three GEO datasets.

Four GSE datasets of CCA were retrieved form GEO including GSE26566, GSE32225, GSE76297 and GSE89749. Of these, GSE89749 which comprised of a small number of normal cases and showed the abnormality of gene distribution was excluded from the study. The GSE26566 contained 104 cancer and 59 normal cases, while GSE32225 comprised of 149 cancer and 6 normal cases. The CCA cases that formed both datasets were non-*Opisthorchis viverrini* (*OV*)-associated CCA. GSE76297 datasets consisted of 91 *OV*-associated CCA and 92 surrounding liver tissues. The volcano plot elucidated DEGs of CCA and normal tissues (Figs. 1A–1C). Based on the selection criteria of an adjusted *P* value <0.05 and absolute log fold-changes ≥ 0.5, a total of 2,029, 4,153 and 4,212 DEGs were identified from GSE26566, GSE32225 and GSE76297. 1,152 and 877 genes were up- and down-regulated in the GSE26566 dataset. 2,165 and 1,988 genes were up- and down-regulated in GSE32225. 2,407 upregulated and 1,805 down-regulated genes were identified in GSE76297 dataset. Finally, Venn diagram analysis revealed that there are 226 common DEGs in the three microarray datasets, in which 124 were up-regulated and 102 down-regulated genes (Figs. 1D and 1E).

### Gene ontology and pathway enrichment analysis

**Figure 2:**
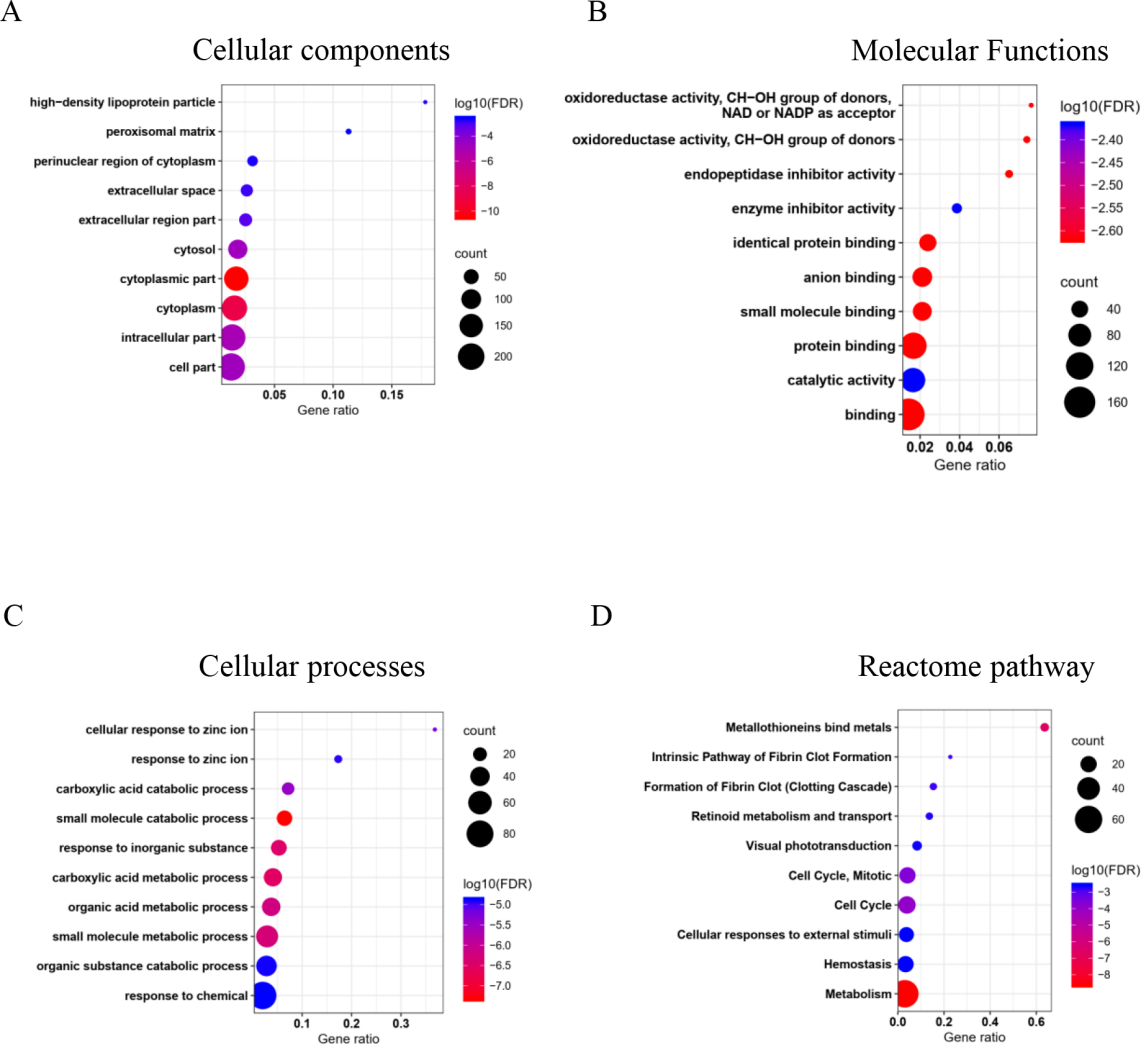
Top 10 gene ontology (GO) and reactome pathway enrichment analyses of 226 common DEGs identified from three GEO datasets. The common DEGs are categorized by GO and pathway enrichment analysis into (A) cellular components, (B) molecular function, (C) cellular processes, and (D) reactome pathways. The *x*-axis demonstrates gene ratio. The pseudo color from red to blue represents the false discovery rate, from the lowest to the highest, respectively. The circle sizes indicate the gene count.

The GO and pathway enrichment analysis of common DEGs elucidated in cellular component (CC) terms (Fig. 2A), common DEGs were mainly enriched in cytoplasm, extracellular space, high-density lipoprotein particles and in the peroxisomal matrix. The common DEGs were also involved with several molecular functions (MF) (Fig. 2B) such as oxidoreductase activity, endopeptidase inhibitor activity and enzyme inhibitor activity. In cellular process terms (Fig. 2C), the results showed that common DEGs were significantly enriched in carboxylic acid catabolic processes, whereas small molecule metabolic processes, were responsive to inorganic substances and organic acid metabolic processes. Moreover, the Reactome pathway enrichment analysis indicated that common DEGs were mainly enriched in metabolism, metallothionein bound metals and cell cycles, especially mitosis (Fig. 2D).

### PPI of common DEGs and hub gene identifications

**Figure 3:**
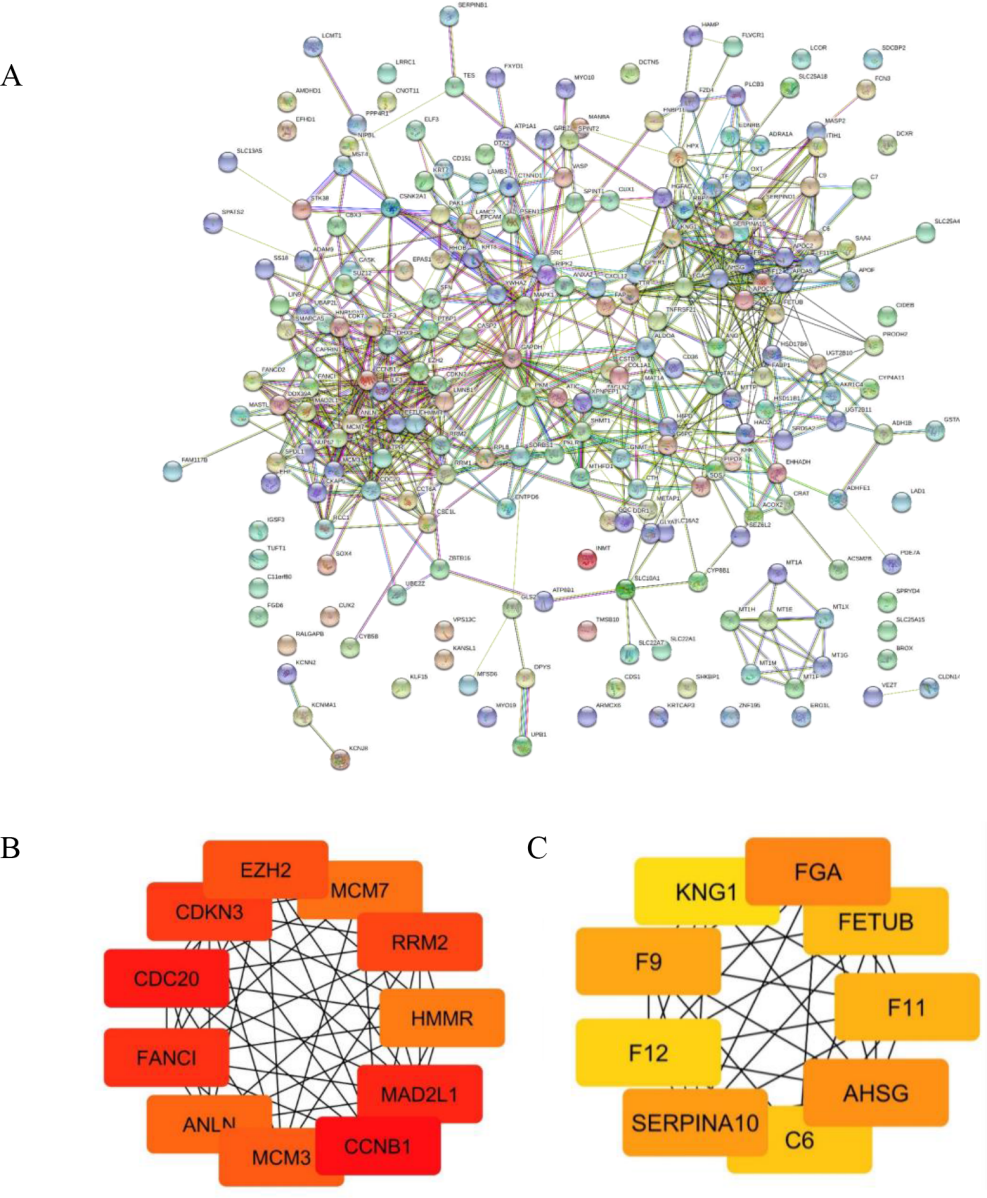
Protein-protein interaction (PPI) network of common DEGs and Module analysis. (A) STRING protein-protein interaction network of 226 common DEGs identified from three GEO datasets. (B–C) Subnetwork of top 20 hub genes from protein-protein interaction network using Cytoscape software. Node color reflects degree of connectivity. The pseudocolor scale from red to yellow represents the top nine hub genes rank from 1–20. Red color represents highest degree, and orange color represents intermedia degree, and yellow color represents lowest degree.

The PPIs of 226 common DEGs were constructed using STRING software as shown in (Fig. 3A). The hub genes based on the PPI network were further prioritized (Figs. 3B and 3C). The results categorized the top 20 hub genes automatically into two groups. The up-regulated hub genes from common DEGs, including CCNB1, CDC20, MAD2L1, FANCI, CDKN3, RRM2, EZH2, MCM3, ANLN, MCM7 and HMMR were ranked from the degree of connectivity with other proteins (binding scores) (Table 1). Down-regulated hub genes included FGA, AHSG, SERPINA10, F9, F11, FETUB, C6, F12 and KNG1. Node colors are represented for the degree of connectivity. The pseudocolor scale from red to yellow represents the gene ranking from 1 to 20. The dark red color represents the highest degree while an orange color stands for the intermediate degree and yellow color is the lowest degree. Most of the up-regulated hub genes were involved with cell division and cell cycle control, nucleotide metabolism and cell movement.

**Figure 4:**
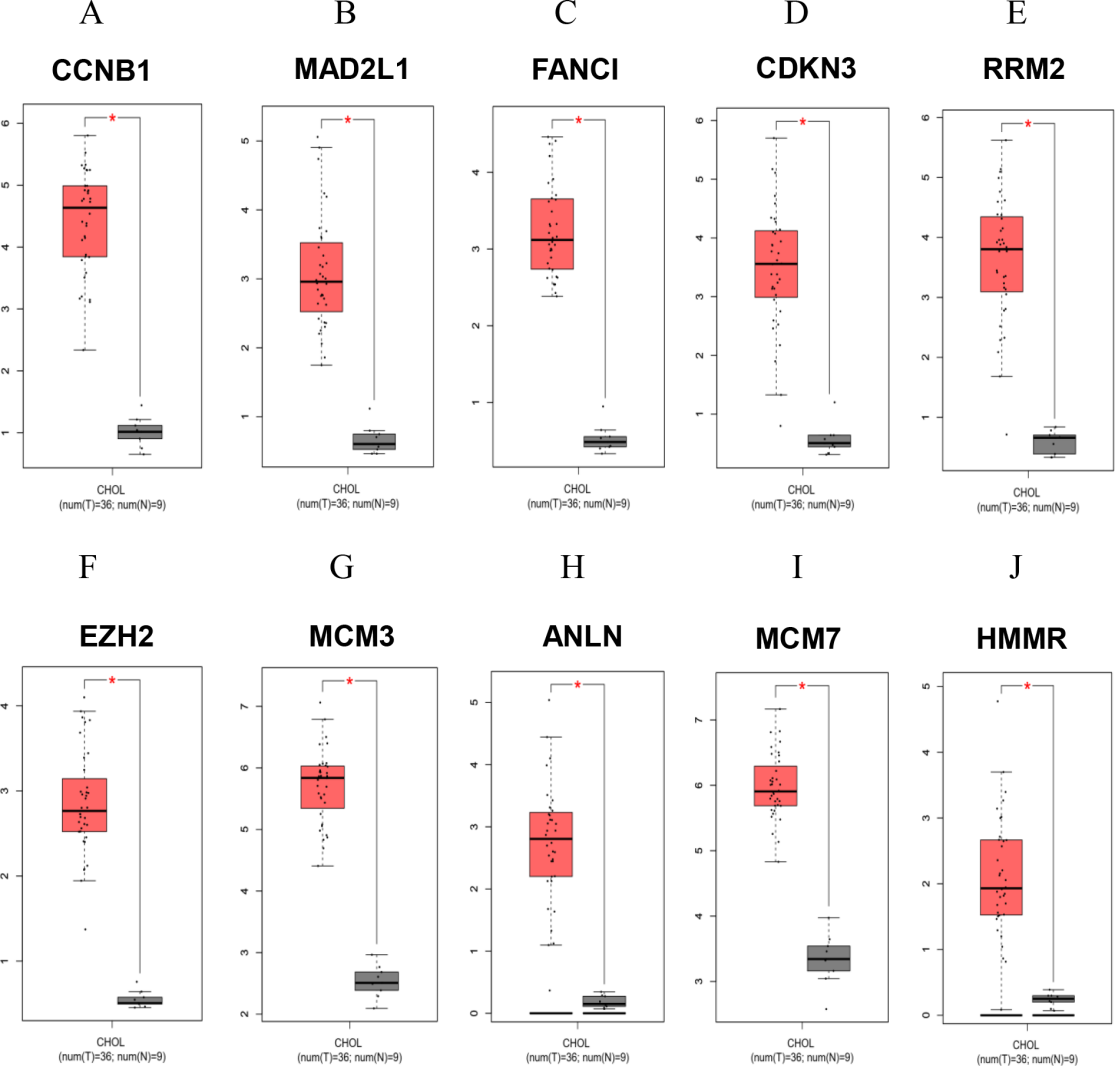
Significantly expressed 10 genes in CCA and normal bile duct epithelium tissues. Box plots analyses compared the expression levels of the specified genes in patient CCA tissues (red) and normal adjacent tissues (grey). The data were analyzed from The Cancer Genome Atlas (TCGA) databases using GEPIA (Gene Expression Profiling Interactive Analysis), a web-based tool. *P < 0.05.

**Figure 5:**
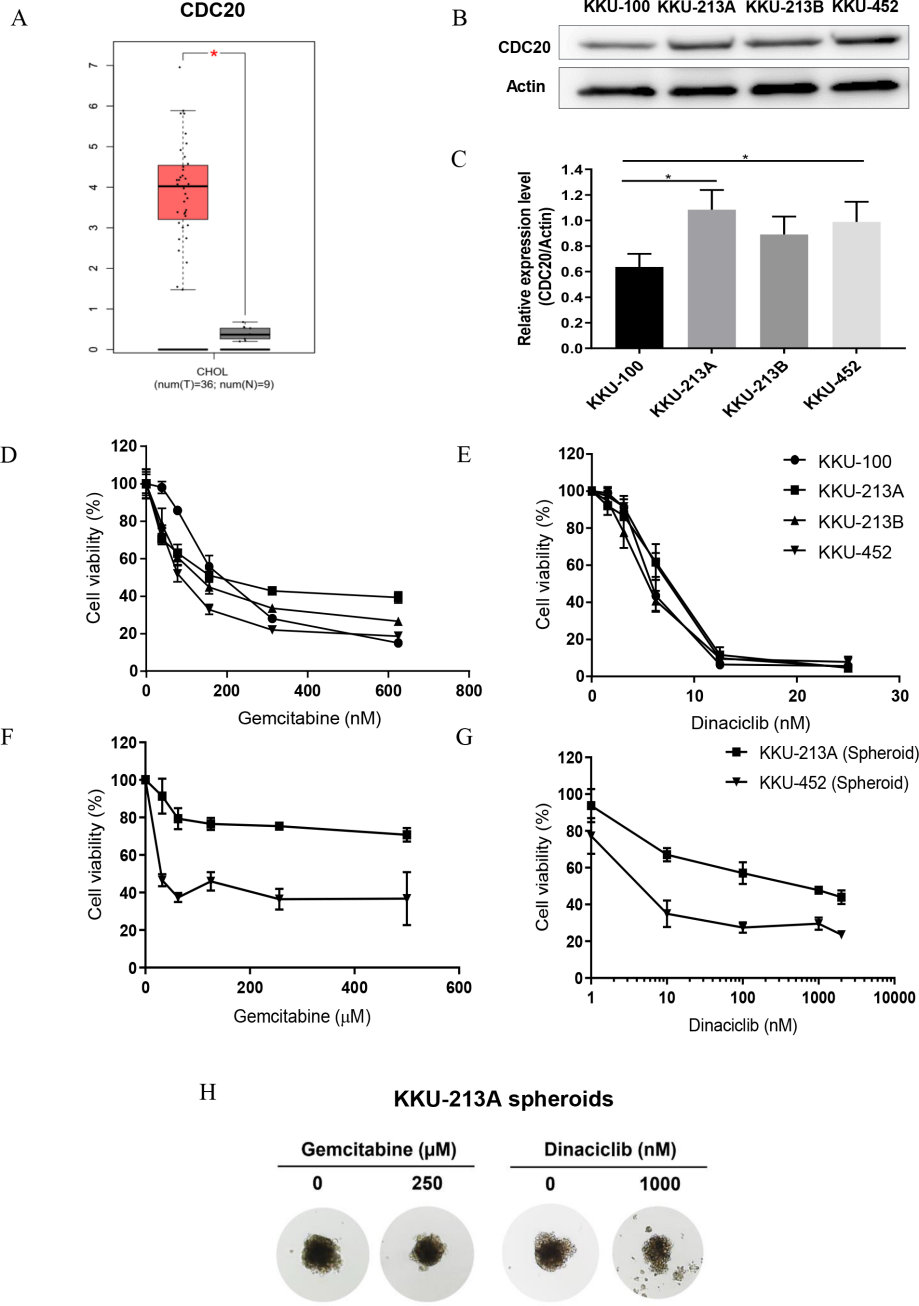
Anti-tumor activities of dinaciclib and gemcitabine in CCA cell lines. (A) The expression levels of CDC20 mRNA in patient CCA tissues (red) and normal counterparts (grey) retrieved from TCGA. (B) Representative western blots of CDC20 in four CCA cell lines and (C) the semi-quantitative analysis. Anti-tumor activities of dinaciclib and gemcitabine in CCA cell lines were determined in (D–E) 2D and (F–G) 3D cell culture model. (H) The representative multicellular tumor spheroids of KKU-213A after 72 h incubation with or without the indicated drugs. *P < 0.05.

To confirm the reliability of the analyzed results, the expression levels of all up-regulated hub genes using different datasets and different databases were further examined. RNA sequencing profiles were retrieved from The Cancer Genome Atlas (TCGA, https://www.cancer.gov/about-nci/organization/ccg/research/structural-genomics/tcga). Box plots were plotted for the elucidation expression level of the hub genes in patient CCA tissues compared with the normal tissues (Figs. 4 and 5A). The results elucidated strong evidence to support the analysis that all the identified hub genes were highly expressed in CCA when compared with normal controls even though the analysis was performed on different databases and different platforms.

### Prioritizing and prediction of a novel targeted drug for CCA

Novel candidate drugs targeted for CCA were based on 11 up-regulated hub genes using PanDrugs, a bioinformatics platform, to prioritize anticancer drug treatments. PanDrugs not only provided drug status descriptions but it also predicted the possible drug response and the interaction between drugs. The drug prediction and prioritizing in Table 2 shows several potential novel drugs for 6 of the 11 upregulated hub genes (Fig. 3B). CDC20 was the candidate that fit to the selection criteria (Fig. S1) as CDC20 plays significant roles in cell cycle and mitosis, and was the high-scoring hub gene (Fig. 3B). There are several candidate drugs suggested for CDC20 (Table 2). Dinaciclib, however, was selected to be the drug of choice for CDC20 in this study as it has never been reported for CCA, and the safety and efficacy has been reported in the clinical trial phase III (Ghia et al., 2015; Sharp & Corp, 2017).

### CDC20 is a candidate novel target for CCA

After the in-silico analysis, a series of experiments for investigating the antitumor activity of dinaciclib in CCA cell lines under 2D and 3D cell culture models was performed. CDC20 mRNA and protein were investigated in four CCA cell lines, KKU-100, KKU-213A, KKU-213B and KKU-452, using real-time PCR and western blot analysis. Expression of CDC20 in patient CCA tissues was significantly higher than those of normal counterpart (Fig. 5A), and all CCA cell lines differentially expressed *CDC20* mRNA (Fig. S2 and File S1) and protein (Figs. 5B and 5C). The anti-tumor activity of dinaciclib in CCA cell lines was compared with that of gemcitabine, the standard chemotherapeutic agent for CCA treatment. In the 2D cell culture model (Figs. 5D and 5E), dinaciclib elucidated very effective anti-tumor activity against CCA cell lines at approximately 40 times of the 50% inhibitory concentrations (IC_50_) lower than gemcitabine (Table 3). In the 3D cell culture model (Figs. 5F and 5G), KKU213A and KKU-452 spheroids were treated with various concentrations of dinaciclib or gemcitabine for 72 h. The results demonstrated that dinaciclib had highly effective antitumor activity better than a thousand times gemcitabine in both CCA cell lines (Table 3). The IC_50_ of KKU-213A and KKU-452 spheroids to dinaciclib were 514.8 nM and 4.7 nM, whereas the IC_50_ of gemcitabine against KKU-213A spheroid cannot be calculated because it was higher than 500 ×10^3^ nM, the maximum tested drug concentration. A similar trend was observed for KKU-452 spheroids with gemcitabine IC_50_ is 25.78 ×10^3^ nM. The KKU-213 spheroid morphology at 72 h exposure time with both drugs and vehicle were demonstrated in Fig. 5H. It was then concluded that the CCA cells were more sensitive to dinaciclib than gemcitabine.

## Discussion

The identification of the molecular mechanisms of cancer cells is crucial for diagnosis and therapy of cancer patients. Various high throughput screening (HTS) techniques, cDNA microarray and RNA sequencing, are widely used to explore DEGs involved in carcinogenesis and progression which has provided valuable information for clinical applications (*Lowe et al., 2017*). A huge amount of corresponding data from a cDNA microarray and RNA sequencing is stored in several public databases such as ENCODE (https://www.encodeproject.org/), TCGA, ICGC (https://dcc.icgc.org/) and GEO. Integration of multiple HTS datasets (cDNA microarray and RNA sequencing) is considered a better approach of enhancing the reliability of results than an individual HTS dataset (*Yan et al., 2018; Le, 2019; Huang et al., 2019*).

**Table 1:** Functional roles of top 20 hub genes in CCA based on transcriptomic data.

Rank	Name	Hub score	Expression	Function
1	CCNB1	138571	Up	The protein encoded by this gene is a regulatory protein involved in mitosis.
2	CDC20	138182	Up	CDC20 appears to act as a regulatory protein interacting with several other proteins at multiple points in the cell cycle.
3	MAD2L1	134648	Up	MAD2L1 is a component of the mitotic spindle assembly checkpoint that prevents the onset of anaphase until all chromosomes are properly aligned at the metaphase plate.
4	FANCI	131160	Up	Fanconi anemia is a genetically heterogeneous recessive disorder characterized by cytogenetic instability, hypersensitivity to DNA crosslinking agents, increased chromosomal breakage, and defective DNA repair.
5	CDKN3	130518	Up	The gene was identified as a cyclin-dependent kinase inhibitor, and has been shown to interact with, and dephosphorylate CDK2 kinase, thus prevent the activation of CDK2 kinase.
6	RRM2	129534	Up	This gene encodes one of two non-identical subunits for ribonucleotide reductase.
7	EZH2	123630	Up	This gene encodes a member of the Polycomb-group involved in maintaining the transcriptional repressive state of genes over successive cell generations.
8	MCM3	89514	Up	Involved in the initiation of eukaryotic genome replication.
9	ANLN	87121	Up	This gene encodes an actin-binding protein that plays a role in cell growth and migration, and in cytokinesis.
10	MCM7	49994	Up	The protein encoded by this gene is one of the highly conserved mini-chromosome maintenance proteins (MCM) that are essential for the initiation of eukaryotic genome replication.
11	HMMR	46968	Up	The protein encoded by this gene is involved in cell motility.
12	FGA	25564	Down	This gene encodes the alpha subunit of the coagulation factor fibrinogen.
13	AHSG	25254	Down	It is involved in several processes, including endocytosis, brain development, and the formation of bone tissue. Defects in this gene are a cause of susceptibility to leanness.
14	SERPINA10	23448	Down	It inhibits the activity of coagulation factors Xa and XIa in the presence of protein Z, calcium and phospholipid.
15	F9	23304	Down	This gene encodes vitamin K-dependent coagulation factor IX that circulates in the blood as an inactive zymogen.
16	F11	21072	Down	This gene encodes coagulation factor XI of the blood coagulation cascade.
17	FETUB	17683	Down	The protein encoded by this gene is a member of the fetuin family, part of the cystatin superfamily of cysteine protease inhibitors.
18	C6	15364	Down	This gene encodes a component of the complement cascade.
19	F12	11646	Down	This gene encodes coagulation factor XII which circulates in blood as a zymogen.
20	KNG1	6769	Down	This gene uses alternative splicing to generate two different proteins- high molecular weight kininogen (HMWK) and low molecular weight kininogen (LMWK). HMWK is essential for blood coagulation and assembly of the kallikrein-kinin system.

In the present study, an in-silico analysis using several bioinformatics approaches for screening and identification novel molecular targets for CCA treatment was performed using three microarray datasets of patient CCA tissues. The analyses revealed several novel targets for CCA treatment. Of these, CDC20, a regulatory protein involves in multi-cell cycle checkpoints was identified as a novel target molecule for CCA treatment and dinaciclib, a pan CDK inhibitor, was suggested as a compatible drug for CDC20. The suggestion from the in-silico analyses was proved by the in vitro cytotoxic experiments of dinaciclib in comparison with Gemcitabine in human CCA cell lines.

Three microarray datasets from CCA patients including GSE26566, GSE32225 and GSE76297 were retrieved from the GEO database and used in this study. From GEO2R and Venn diagram analysis, a total of 226 common DEGs including 124 up-regulated and 102 down-regulated genes in CCA tissues were revealed. GO and pathway enrichment analysis revealed that common DEGs were enriched in metabolism, and cell cycles especially in the mitosis phase. As is well known, one of the hallmarks of cancers is the alteration of metabolism in which cancer cells predominantly use the glycolytic pathway as the main energy source rather than tricarboxylic acid cycle (TCA) even in an adequate oxygen condition, known as “Aerobic glycolysis” or the “Warburg effect” (*Warburg, 1956; Schwartz, Supuran & Alfarouk, 2017*). The understanding of the cancer metabolism also provides an opportunity for development specific novel targets for cancer diagnosis and treatment. At present, several alterations of glycolytic related molecules have been reported in CCA such as glucose transporters (GLUT) hexokinase (HK) II (Paudyal et al., 2008; Thamrongwaranggoon et al., 2017), and Tumor M2-pyruvate kinase (PKM2) (*Cuenco et al., 2018*). Moreover, the alteration in expression levels of other molecules such, c-MET, RAS/BRAF, EGFR/ERBB2 which are involved downstream, affect cancer cell proliferation and development. (*Terada, Nakanuma & Sirica, 1998; Miyamoto et al., 2011; Churi et al., 2014; O’Dell et al., 2012; Leone et al., 2006; Ito et al., 2001*).

**Table 2:** The potentially druggable targets and prioritized drugs identified for CCA treatment using PanDrugs software.

**Gene(s)**	**Show Drug Name**	**Status Description**	**Therapy**	**Drug response**	**Best Interaction**
RRM2	CLADRIBINE	Approved for blood cancer	CHEMOTHERAPY	SENSITIVITY	direct-target
RRM2	CLOFARABINE	Approved for blood cancer	CHEMOTHERAPY	SENSITIVITY	biomarker
RRM2	HYDROXYUREA	Approved for blood cancer	CHEMOTHERAPY	SENSITIVITY	biomarker
EZH2	DABRAFENIB	Approved for skin cancer	TARGETED_THERAPY	SENSITIVITY	biomarker
RRM2	FLUDARABINE	Approved for blood cancer	CHEMOTHERAPY	SENSITIVITY	biomarker
RRM2	FLUDARABINE PHOSPHATE	Approved for blood cancer	CHEMOTHERAPY	SENSITIVITY	biomarker
CDC20—CDKN3	AT7519	Clinical Trials	–	SENSITIVITY	pathway-member
RRM2	MOTEXAFIN GADOLINIUM	Clinical Trials	–	SENSITIVITY	direct-target
RRM2	IMEXON	Clinical Trials	–	SENSITIVITY	direct-target
CDC20	DINACICLIB	Clinical Trials	–	SENSITIVITY	pathway-member
CDC20	FLAVOPIRIDOL	Clinical Trials	–	SENSITIVITY	pathway-member
CDC20	BAY1000394	Clinical Trials	–	SENSITIVITY	pathway-member
CCNB1—EZH2	SELUMETINIB	Clinical Trials	–	BOTH?	biomarker
EZH2	1032350 − 13 − 2	Clinical Trials	–	SENSITIVITY	biomarker
RRM2	TRIAPINE	Clinical Trials	–	SENSITIVITY	biomarker
CDKN3	602306 − 29 − 6	Clinical Trials	–	SENSITIVITY	biomarker
CDKN3	PHA-793887	Clinical Trials	–	SENSITIVITY	biomarker
CDKN3	RONICICLIB	Clinical Trials	–	SENSITIVITY	biomarker
HMMR	HYALURONIC ACID	Clinical Trials	–	SENSITIVITY	direct-target
CDC20	ALSTERPAULLONE	Experimental	–	SENSITIVITY	pathway-member
CDC20	HYMENIALDISINE	Experimental	–	SENSITIVITY	pathway-member
CDC20	INDIRUBIN-3′-MONOXIME	Experimental	–	SENSITIVITY	pathway-member
CDC20	OLOMOUCINE	Experimental	–	SENSITIVITY	pathway-member
CDC20	SU9516	Experimental	–	SENSITIVITY	pathway-member
EZH2	A-395	Experimental	–	SENSITIVITY	biomarker
EZH2	GSK126	Experimental	–	SENSITIVITY	biomarker
RRM2	TEZACITABINE	Experimental	–	SENSITIVITY	biomarker
FANCI	LY2183240	Experimental	–	SENSITIVITY	biomarker
FANCI	SB 225002	Experimental	–	SENSITIVITY	biomarker

The PIP network and module analysis in the currently study demonstrated the top 20 high-scoring hub genes in CCA. Several of these identified hub genes, for instance, CCNB1, CDC20, MAD2L1, were involved in mitosis and cell cycle control (Table 1) (*Percy et al., 2000; Chi et al., 2019; Zhang et al., 2019; Wang et al., 2015*). The computational analysis for prioritizing and predicting of novel targeted drugs based on the 11 up-regulated hub genes by PanDrugs suggested several new targets and drug of choices. Among these, CDC20 is the most interested target as: firstly, it was upregulated in patient CCA tissues compared with the normal counterpart tissues (Fig. 5A). Secondly, it plays an important role in chromosome segregation (Kapanidou, Curtis & Bolanos-Garcia, 2017) during metaphase-anaphase transition (*Fujimitsu, Grimaldi & Yamano, 2016; Wang et al., 2015*) and also other cellular processes, for example suppressing apoptosis (*Harley et al., 2010; Wan et al., 2014*). Thirdly, expression of CDC20 have been demonstrated to be involved with poor patient outcomes in several human malignancies. For instance, the high expression of the CDC20 gene were associated with poor prognosis and outcome of breast cancer patients (*Karra et al., 2014*). Overexpression of CDC20 in tumor tissues was related with poor differentiation and a lower 5-year recurrence-free survival rate of pancreatic cancer patients (*Li et al., 2003; Chang et al., 2012)*, and a short survival of colorectal cancer (*Wu et al., 2013*). Moreover, knockdown of CDC20 gene inhibited cell growth and induced the G2/M arrest in cell cycle of lung cancer cells (*Kidokoro et al., 2008*). Lastly, several agents affected CDC20 as the pathway-member are approved for clinical trials or under experiments (Table 2).

**Table 3:** Comparison the anti-tumor activity of dinaciclib and gemcitabine in CCA cell lines cultured under 2D and 3D cell culture models.

**2D cell culture models**	**IC50 (nM)**	
**CCA cell lines**	**Gemcitabine**	**Dinaciclib**
KKU-100	193 ± 16	5.82 ± 0.42
KKU-213A	241 ± 13	7.37 ± 0.37
KKU-213B	146 ± 30	5.43 ± 0.42
KKU-452	87 ± 3	6.93 ± 0.38
**3D cell culture models**	**IC50 (nM)**	
**CCA cell lines**	**Gemcitabine**	**Dinaciclib**
KKU-213A	>500 ×10^3^	514.8 ± 139
KKU-452	25.78 ± 8.36 ×10^3^	4.70 ± 1.20

Dinaciclib treatment effectively suppressed tumor growth in CCA cell lines cultured under 2D and 3D cell culture models (Figs. 5D–5G). In similar in-vitro studies, dinaciclib elucidated antitumor activity against cultured cells in 2D models at low nanomolar levels such as, 12, 8, 17 and 14 nM IC_50_ for prostate, breast, colon, and ovarian cancer cell lines (*Parry et al., 2010*). Interestingly, the tumor-suppressing effect of dinaciclib was better than gemcitabine, the current standard treatment drugs for CCA. The 4 CCA cell lines tested showed different response to gemcitabine as indicated by a wide IC_50_ range of 87–241 nM, whereas all cell lines showed a similar sensitivity to dinaciclib with a narrow IC_50_ range of 5.4–6.9 nM. Cancers seem to be more sensitive to dinaciclib than gemcitabine as the similar observations were also reported in several pancreatic cancer cell lines (*Khan et al., 2020*) and hepatocellular carcinoma (*Shao et al., 2019; Hassan et al., 2019*). The different response of cancer cells to gemcitabine and dinaciclib may be due to the different drug targets and actions. Gemcitabine targets the DNA synthesis and induces cell death, whereas dinaciclib acts on several CDKs in cell cycle and hence targets several points of the cell cycle at the same time. Moreover, as the 4 CCA cell lines were established from different subtypes of CCA, using dinaciclib as the treatment of choice for CCA may give more advantage than gemcitabine in such the way that dinaciclib exhibited a high anti-tumor activity independently of CCA subtypes.

Dinaciclib is the novel generation of the potent multi-CDKs inhibitor which can suppress CDK1, CDK2, CDK5 and CDK9 (*Parry et al., 2010*) with the IC_50_ in a low nanomolar range (1–4 nM). The effective anti-tumor activity of Dinaciclib have been elucidated both in vitro and in vivo in various types of cancers (*Rello-Varona et al., 2019; Chen et al., 2016*). Enhancing G2/M phase arrest and induction of apoptosis by dinaciclib have been demonstrated in the in vitro and xenografted mouse model in thyroid cancer (*Lin et al., 2017*) and the triple negative breast cancer (*Rajput et al., 2016*). In addition, dinaciclib was shown to trigger abnormal mitotic division (anaphase catastrophe) in lung cancer cells through, CDK1 and CDK2 suppression (*Danilov et al., 2016*). Moreover, dinaciclib has been used in clinical trials of several cancers, such as chronic lymphocytic leukemia (*Flynn et al., 2015; Gojo et al., 2013*) and breast cancer (*Mita et al., 2014*). The tolerant side effects such as transient gastrointestinal toxicities, liver function tests abnormalities, fatigue, and hypotension have been reported (*Gojo et al., 2013*).

A similar but different approach has been performed using a dataset from GEO (GSE26566) to find the potential candidate treatment agents for CCA without validation (*Chujan et al., 2018*). Recently, Ye et al. (2020) has identified the key genes associated with the progression of intrahepatic CCA using three GEO dataset including GSE107943, GSE119336 and GSE26566. The present study provided several target candidates different from those reported in the previous studies. This may be due to the different objectives, different workflow of the analysis and different GEO dataset used. Combining various bioinformatics methods using several gene expression datasets may reduce the bias and strengthen the analysis outcome. The experimental evidence in vitro and in vivo must be conducted to confirm the analysis outcome before translating to the clinical practice.

Taken together, the current study provided several novel draggable-target molecules for CCA treatment from computational analysis. The reliability of in-silico results for biological investigation was confirmed in CCA cell lines. The compatible results between computational and biological investigations demonstrated very interesting and strong evidence that all identified hub genes, especially CDC20 was highly expressed in CCA tissues and may be used as an effective novel target for CCA treatment.

## Conclusions

In the present study, the aim was to identify a novel molecular target for CCA treatment using bioinformatics analysis based on three transcriptomic datasets available in the GEO database. The identified hub genes were mostly involved with metabolism, mitosis and cell cycle control which indicated that, CCA was highly effective in these processes. CDC20 was suggested by bioinformatic analysis as a potential novel target for CCA. Not only using in-silico analysis, the expression levels of CDC20 in CCA cell lines as well as the investigation of the anti-tumor activity of novel potential targeted drugs against CCA cells were compared with the standard chemotherapy, gemcitabine. This novel drug has a stronger antitumor activity than gemcitabine.

##  Supplemental Information

10.7717/peerj.11067/supp-1 Supplemental Information 1 Realtime PCR raw data

10.7717/peerj.11067/supp-2 Supplemental Information 2 Raw data from 2D-ATP assay in CCA cell line with Dinaciclib and Gemcitabine

10.7717/peerj.11067/supp-3 Supplemental Information 3 Raw data from 3D-ATP assay in CCA cell line with Dinaciclib and Gemcitabine

10.7717/peerj.11067/supp-4 Supplemental Information 4 Cholangiocarcinoma TCGA PanCancer raw data

10.7717/peerj.11067/supp-5 Supplemental Information 5 List of common up and down-regulated DEGs

10.7717/peerj.11067/supp-6 Supplemental Information 6 DEGs data from GEO2R

10.7717/peerj.11067/supp-7 Supplemental Information 7 Fig. 5H raw data

10.7717/peerj.11067/supp-8 Supplemental Information 8 Workflow for bioinformatics and in vitro analysis

10.7717/peerj.11067/supp-9 Supplemental Information 9 Expression levels of CDC20 mRNA in CCA cell lines

## Additional Information and Declarations

### Data Availability

The following information was supplied regarding data availability: Raw measurements are available in the Supplemental Files.
