# Supplementary figures and images for: Bioinformatics analysis identified CDC20 as a potential drug target for cholangiocarcinoma
Source: PeerJ. 2021 Mar 17;9:e11067. doi: 10.7717/peerj.11067 (PMC7980698; doi:10.7717/peerj.11067)

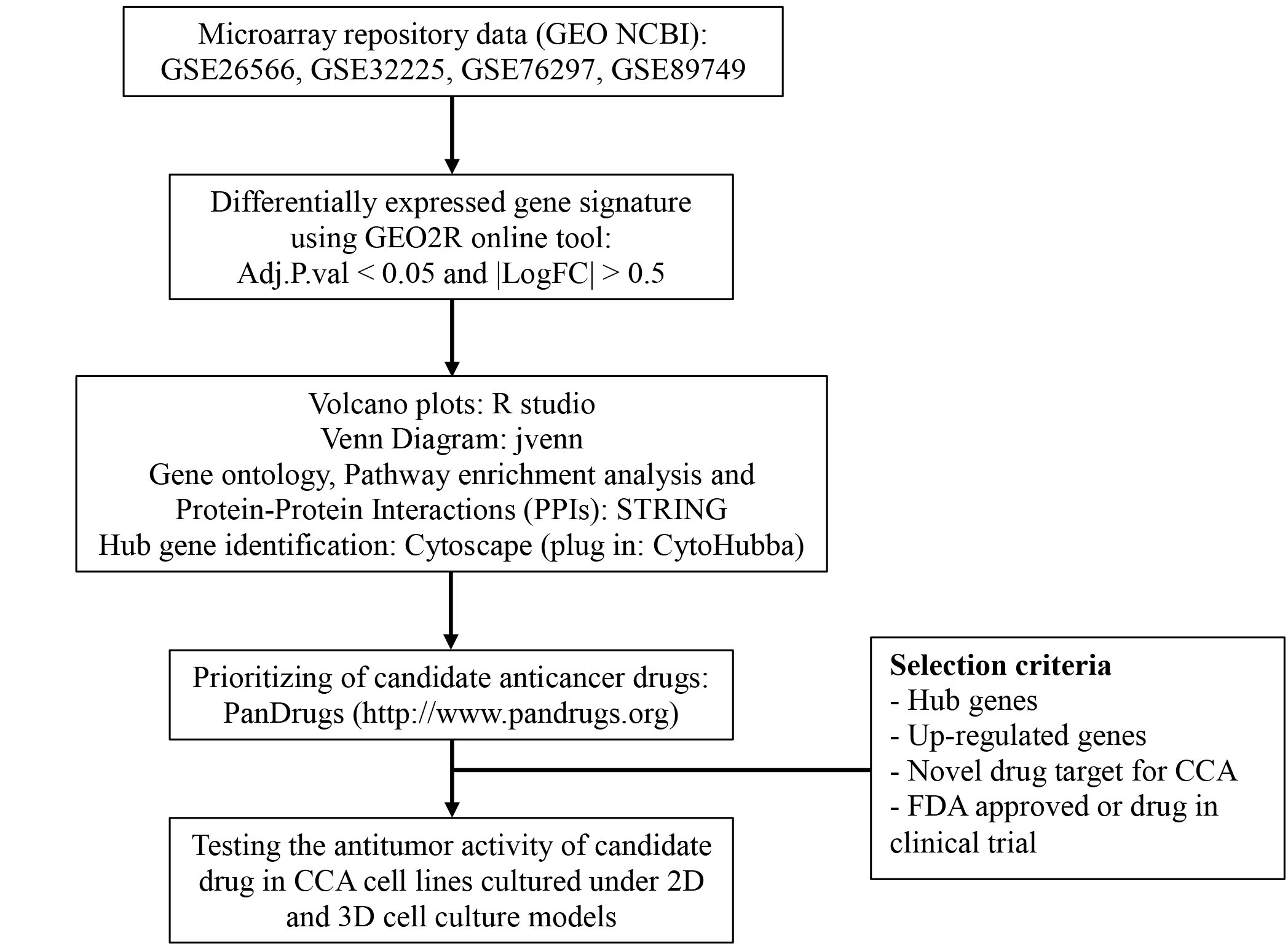

Supplement: Supplemental Information 8 [file peerj-09-11067-s008.jpg]

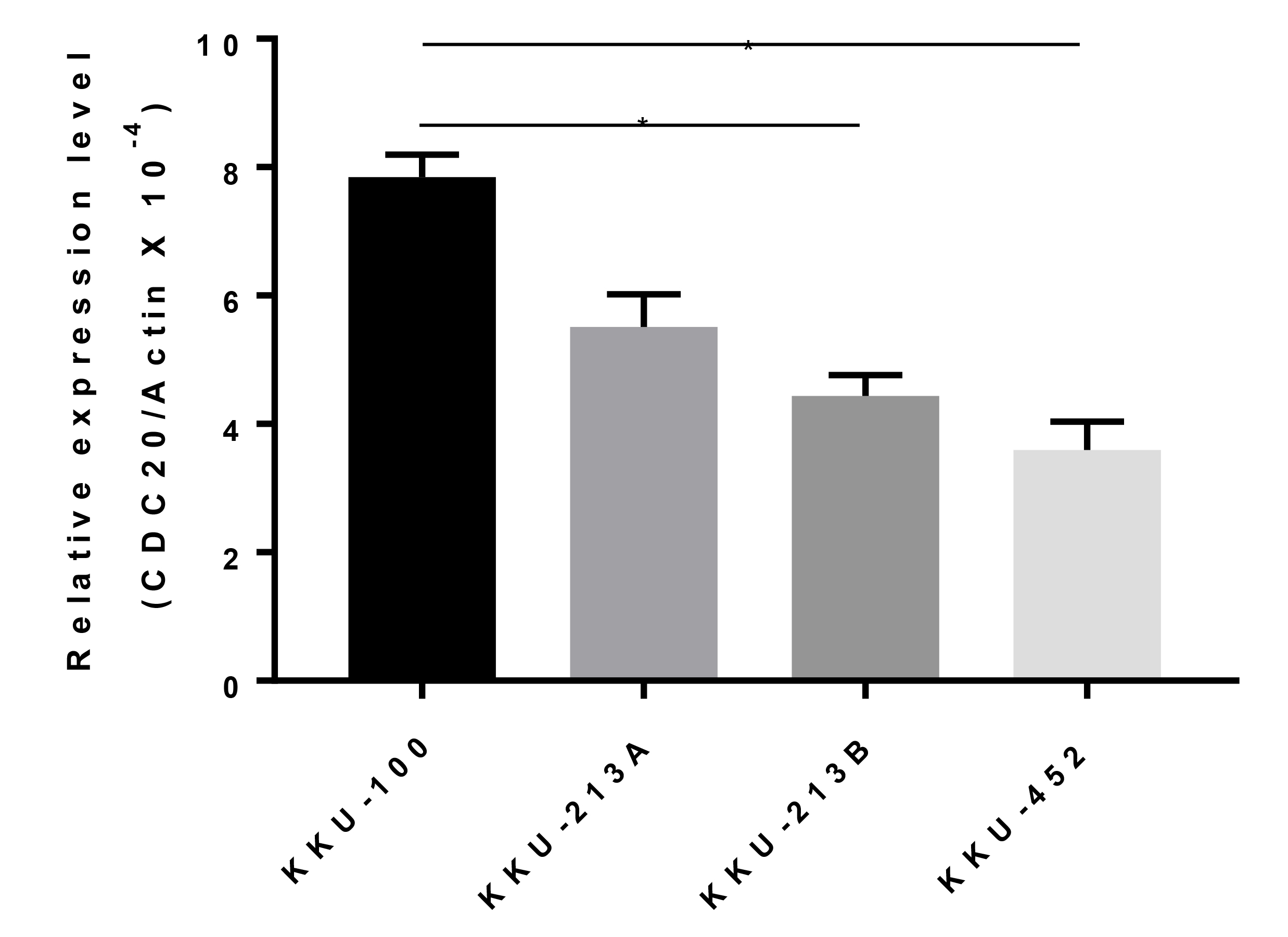

Supplement: Supplemental Information 9 [file peerj-09-11067-s009.jpg]
